# Altered resting perfusion and functional connectivity of default mode network in youth with autism spectrum disorder

**DOI:** 10.1002/brb3.358

**Published:** 2015-06-25

**Authors:** Kay Jann, Leanna M Hernandez, Devora Beck-Pancer, Rosemary McCarron, Robert X Smith, Mirella Dapretto, Danny J J Wang

**Affiliations:** 1 Laboratory of FMRI Technology (LOFT), Ahmanson-Lovelace Brain Mapping Center, Department of Neurology, University of CaliforniaLos Angeles, California; 2 Department of Psychiatry and Biobehavioral Sciences, University of CaliforniaLos Angeles, California

**Keywords:** arterial spin labeling, autism spectrum disorder, cerebral blood flow, default mode network, dorsal ACC, functional connectivity

## Abstract

**Background:**

Neuroimaging studies can shed light on the neurobiological underpinnings of autism spectrum disorders (ASD). Studies of the resting brain have shown both altered baseline metabolism from PET/SPECT and altered functional connectivity (FC) of intrinsic brain networks based on resting-state fMRI. To date, however, no study has investigated these two physiological parameters of resting brain function jointly, or explored the relationship between these measures and ASD symptom severity.

**Methods:**

Here, we used pseudo-continuous arterial spin labeling with 3D background-suppressed GRASE to assess resting cerebral blood flow (CBF) and FC in 17 youth with ASD and 22 matched typically developing (TD) children.

**Results:**

A pattern of altered resting perfusion was found in ASD versus TD children including frontotemporal hyperperfusion and hypoperfusion in the dorsal anterior cingulate cortex. We found increased local FC in the anterior module of the default mode network (DMN) accompanied by decreased CBF in the same area. In our cohort, both alterations were associated with greater social impairments as assessed with the Social Responsiveness Scale (SRS-total T scores). While FC was correlated with CBF in TD children, this association between FC and baseline perfusion was disrupted in children with ASD. Furthermore, there was reduced long-range FC between anterior and posterior modules of the DMN in children with ASD.

**Conclusion:**

Taken together, the findings of this study – the first to jointly assess resting CBF and FC in ASD – highlight new avenues for identifying novel imaging markers of ASD symptomatology.

## Introduction

Autism spectrum disorders (ASD) are characterized by impairments in social communication, the presence of restricted interests and repetitive behaviors and/or sensory over-responsivity (American Psychiatric Association, 2013). During the past few decades, neuroimaging studies have provided new insights into the neurobiological underpinnings of these behavioral impairments and have revealed aberrant patterns of brain activity in virtually all nodes of the ‘social brain’ in ASD (Hernandez et al. 2014). In addition to altered activation patterns during tasks, changes in functional connectivity (FC) of several key resting brain networks (Assaf et al. 2010; Rudie et al. 2012a,b; Lynch et al. 2013; Hernandez et al. 2014; Maximo et al. 2014; Washington et al. 2014) have been identified, leading to the hypothesis that ASD might be caused by increased (hyper-) or decreased (hypo-) connectivity within specific networks (Kennedy and Courchesne 2008b). One major resting brain network, the default mode network (DMN) has become a focus of research, as it is relevant for self-referential thought, social and emotional processes, and theory of mind (ToM) (Buckner et al. 2008). Existing literature on DMN connectivity in individuals with ASD show increased FC within the frontal lobe, including the medial prefrontal cortex (mPFC), and reduced long-range connectivity from mPFC to posterior cingulate cortex (PCC) and precuneus compared to typically developing (TD) children (Kennedy and Courchesne 2008a; Assaf et al. 2010; Rudie et al. 2012a,b; Lynch et al. 2013; Rudie and Dapretto 2013; Hernandez et al. 2014; Maximo et al. 2014; Washington et al. 2014). Additionally, age-related developmental changes in FC patterns have been reported in children with ASD (Uddin et al. 2013b).

While conventional blood oxygenation level dependent (BOLD) task-related fMRI and resting-state functional MRI (rs-fMRI) are capable of identifying areas of altered task-activity and changes in FC of brain networks, respectively, these methods do not provide a quantitative measure of baseline metabolic activity within these areas and networks. Baseline metabolic activity is, however, another important index of resting brain function. PET and SPECT studies in ASD have assessed baseline metabolic activity by quantifying the cerebral metabolic rate of glucose (CMRglu) and cerebral blood flow (CBF). This line of research has yielded findings suggesting increased global glucose metabolism in male individuals with ASD compared to matched controls, as well as relative hypoperfusion in the frontal and temporal lobe of children with ASD versus a control group of children with intellectual disability (Ohnishi et al. 2000; Boddaert and Zilbovicius 2002; Yang et al. 2011). Due to the use of radioactive tracers, however, PET and SPECT imaging cannot be widely applied in developmental populations such as children with and without ASD.

Arterial spin labeling (ASL) MRI techniques provide a noninvasive alternative to assessing cerebral perfusion by utilizing magnetically labeled arterial blood water as an endogenous tracer. Quantitative ASL measurements of CBF have been validated using 15O-water PET and SPECT (Ye et al. 2000; Xu et al. 2010; Kilroy et al. 2014), and have been shown to provide information comparable to glucose metabolism measured by FDG PET (Newberg et al. 2005). The recent development of pseudocontinuous ASL (pCASL) with background-suppressed (BS) 3D acquisitions (e.g., GRASE – a hybrid of spin and gradient echo and Stack-of-Spirals) has dramatically improved the sensitivity and temporal signal-to-noise ratio (SNR) of this technique (Fernandez-Seara et al. 2008; Alsop et al. 2014), allowing not only reliable CBF measurement but also FC analysis of perfusion image series while minimizing potential BOLD contributions (Chuang et al. 2008; Liang et al. 2012; Dai et al. 2013; Jann et al. 2015a).

These latest developments in ASL open the door to simultaneous assessments of alterations in FC and baseline perfusion/metabolism. The interrelation between these measures may provide meaningful information about aberrant brain activity patterns in clinical populations (Jann et al. 2015a). The purpose of this study was, therefore, to apply cutting edge ASL technology to the examination of cerebral perfusion, functional connectivity, their inter-relation, and their possible associations to ASD symptom severity in a cohort of children with ASD and matched TD controls.

## Methods

### Participants

Seventeen high-functioning children and adolescents with ASD (age [years] mean ± SD: 13.8 ± 2.0; 4f/13 m) and 22 TD participants (12.8 ± 3.6, 3f/19 m) were enrolled in the study. We matched the ASD and TD groups in terms of age, gender, and IQ and confirmed that there were no differences using two-sample *t*-tests or a Chi-squared test with Yates correction for small sample sizes. Clinical diagnosis of ASD was confirmed with the Autism Diagnostic Observation Schedule [ADOS; (Lord et al. 2000)], Autism Diagnostic Interview-Revised [ADI-R; (Lord et al. 1994)] and best clinical judgment. Social functioning of all participants was measured using the Social Responsiveness Scale [SRS, (Constantino et al. 2003)]. Ten children with ASD were not taking medication at the time of the scan, the remaining seven were taking one or a combination of the following: CNS stimulants (4), antihypertensive drugs (2), SSRIs (1), anti-epileptic drugs (1), and norepinephrine reuptake inhibitors (1). Two of the medicated children were on two or more medications. The demographic information for the ASD and TD groups is listed in Table 2010 along with their SRS-total T scores.

**Table 1:** Demographics of study cohort

	ASD	TD	*t*-value	Chi-square	*P*-value
*N*	17	22			
Age	13.8 ± 2.0	12.8 ± 3.6	1.0		n.s.
Gender	4f/13 m	3f/19 m		0.14	n.s.
IQ	107.8 ± 18.7	107.8 ± 14.3	0.01		n.s.
ADOS severity	7.7 ± 1.2	–			
ADOS SA	10.5 ± 2.5	–			
ADOS RRB	2.8 ± 1.7	–			
ADI-R a	19.1 ± 4.5	–			
ADI-R b	15.5 ± 3.3	–			
ADI-R c	6.3 ± 2.6	–			
ADI-R d	3.0 ± 1.1	–			
SRS-total T scores	74.7 ± 13.9	45.9 ± 14.3	6.1		<0.000001

ASD, autism spectrum disorders; TD, typically developing; ADOS, autism diagnostic observation schedule; ADOS SA, Autism Diagnostic Observation Schedule SOCIAL AFFECT; ADOS RRB, Autism Diagnostic Observation Schedule RESTRICTED AND REPETITIVE BEHAVIOR; ADI-R, autism diagnostic interview-revised; SRS, social responsiveness scale

### MRI data acquisition and preprocessing

All MR data were collected on a 3-T Siemens TIM Trio Scanner (Erlangen, Germany) using a 12-channel head coil. An 8-min resting-state perfusion MRI scan was performed using a pCASL sequence with 3D single shot, background-suppressed (BS) GRASE readout (Jann et al. 2015a) with the following parameters: 80 pairs of control and label acquisitions, TR/TE = 3000/22 ms, labeling duration = 1200 ms, postlabeling delay (PLD) = 1000 ms; 26 slices; matrix 64 × 64; voxel 3.44 × 3.44 × 5 mm. In addition, high-resolution anatomical images were measured with a MPRAGE scan (isotropic 1 × 1 × 1 mm^3^ voxels, 176 sagittal slices, 256 × 256 matrix).

Anatomical images were skull stripped and imported into ANTs [Advanced Normalization Tools, (Avants et al. 2009)] to generate a study specific template (Avants et al. 2010). ASL data were first motion corrected for label and control images separately (Wang et al. 2008). ASD and TD groups did not show significant differences in motion parameters (i.e., mean framewise displacement (FD) (Power et al. 2012) for ASD = 0.49 and TD = 0.53; for details see Supplemental Materials). Perfusion images are partly sensitive to BOLD contrast, as image acquisition is based on GRASE readout with a TE of 22 ms. However, this spurious BOLD contrast in the raw ASL image data can be attenuated during subtraction of control and label pairs as well as background suppression. In this study, sinc-subtraction was employed to generate the difference images between label and control image series, which has been shown to minimize BOLD contributions in CBF time series (Aguirre et al. 2002; Liu and Wong 2005). Difference images between label and control images were then converted into CBF time series as well as to a temporal mean CBF-map using a single compartment model accounting for age- and gender-dependent changes in blood T1 (Wu et al. 2010; Jain et al. 2012). CBF time series and mean CBF-maps were coregistered to the individual anatomical MRI and normalized to the study specific template. Finally, normalized images and time series were resliced into MNI space and spatially smoothed with an 8-mm FWHM Gaussian kernel.

### Statistical analysis

To assess perfusion differences between ASD and TD groups, we performed a two-sample two-sided *t*-test with global GM-CBF and age included as covariates (significance set to *P*_voxel_ < 0.05 corrected for type I errors at α_cluster_ < 0.05 using a cluster size threshold [AlphaSim; (Ward 2000)]. Normalized CBF time series were submitted to a group level ICA using GIFT (Calhoun et al. 2001, 2004) to identify functionally connected networks. The ICA model order was estimated by the MDL/BIC criterion (Li et al. 2007). The group-component depicting the DMN was identified by template matching in GIFT and visual inspection of all components. Individual DMN *z*-maps were back-reconstructed using dual regression. These individual maps were then subjected to a one-sample *t*-test (*P* < 0.05 FWE corrected) to compute the overall group DMN. FC strength in ICA is represented by voxels’ *z*-scores that indicate the degree to which a given voxel is integrated within a given network component (i.e., its average connectivity strength to all other voxels in the specific network) (Jann et al. 2015a). Differences in FC between the ASD and TD groups were assessed by a two-sample two-sided *t*-test (*P* < 0.05 corrected for type I errors at α < 0.05 using a cluster size threshold (AlphaSim; (Ward 2000)). In addition, we performed a Seed Based Analysis (SBA) between regions of interest (ROIs) in the mPFC/ACC and the PCC/precuneus. The ROIs were based on the atlas by Power (Power et al. 2011). We selected three ROIs in the posterior brain as these areas are deemed to have distinct connectivity (Lynch et al. 2013), and one in the dorsal ACC/mPFC (Figure S1 and Table S3).

To explore a possible relation between regional CBF and FC, we calculated voxel-wise Pearson correlations between mean CBF maps and DMN *z*-maps across all subjects, as well as within each group separately. The maps resulting from the above analyses were masked with a group DMN map (joint mask of anterior and posterior DMN) to examine effects within this specific network.

### Post hoc region of interest analyses

Using regions where we detected significant group differences in FC and/or CBF, we defined ROIs from which we extracted the individual participant’s values for FC strength (*z*-scores) and regional CBF (mL/100 g/min), respectively. Within these ROIs we assessed the relation between the two parameters of physiological baseline brain function using Pearson correlation (significance at *P* < 0.05). The correlation coefficients for the two groups were compared using Fisher’s *z* transform and test. Finally, a possible association of FC and CBF with symptom severity based on the SRS-total T scores was investigated by Spearman’s rank correlation analyses (significance at *P* < 0.05). In these ROI analyses, regional CBF was corrected for global GM-CBF and age. Furthermore, we repeated the ROI analyses accounting for regional structural variations by including the average Jacobian determinant of the specific ROI as a covariate, which represents the amount of structural expansion or shrinkage during normalization within the ROI (Dubb et al. 2003).

## Results

### Resting CBF differences between ASD and TD

**Figure 1:**
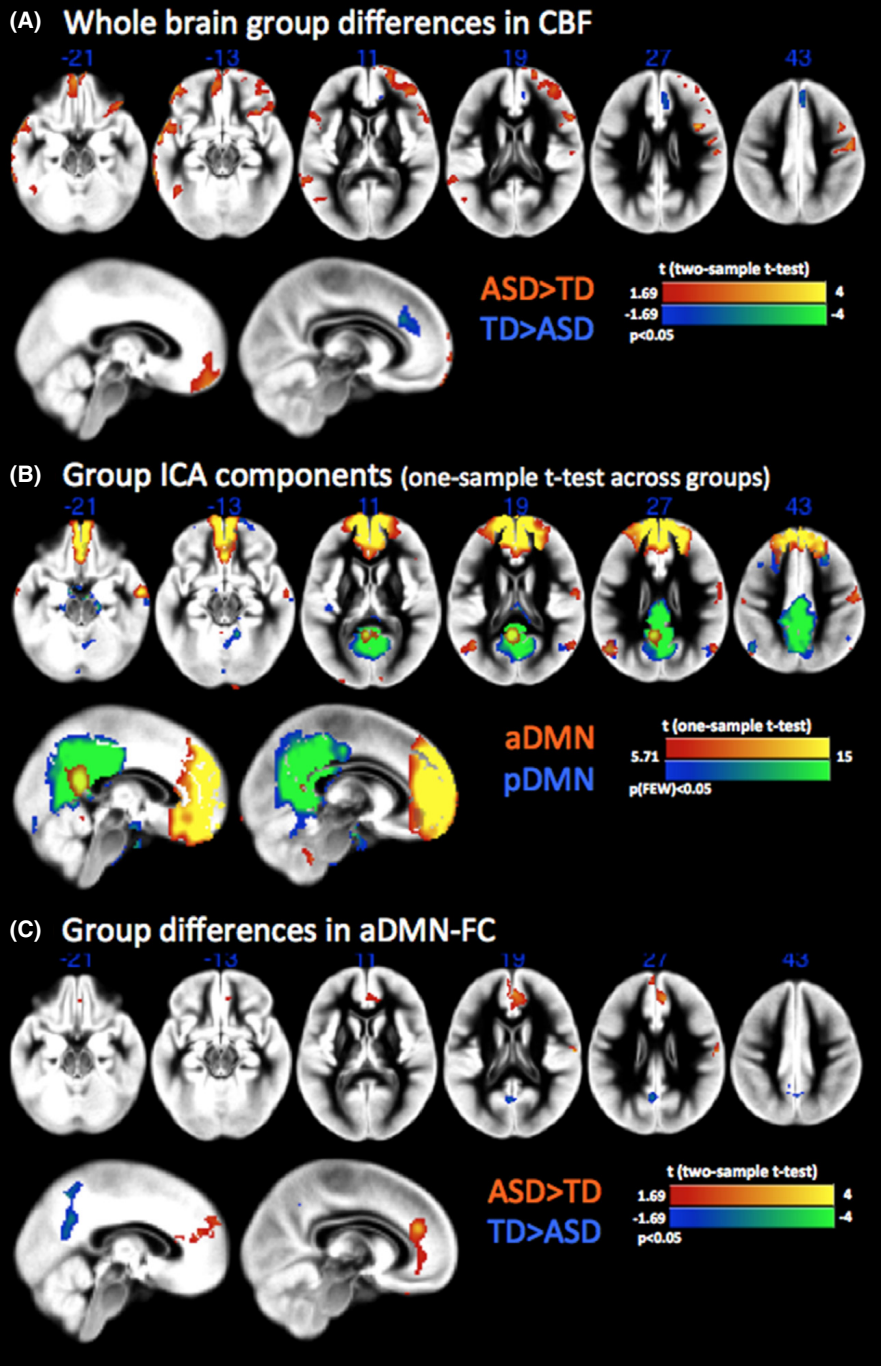
(A) Two-sample two-sided *t*-test revealing areas of hyper- and hypoperfusions in autism spectrum disorders (ASD) versus typically developing (TD). (B) Group default mode network (DMN) components based on Arterial spin labeling (ASL) datasets. Two ICA components were indicating the anterior and posterior modules of the default mode network (DMN), respectively. (C) Two-sample two-sided *t*-test displaying differences in functional connectivity in the aDMN.

Voxel-wise comparison of regional CBF including global GM-CBF and age as covariates revealed areas with significant group differences (*t*_35_ > 1.69, *P* < 0.05, CST_(__α < 0.05)_ = 251 voxels). Children with ASD presented a pattern of widespread hyperperfusion in frontotemporal regions including medial orbitofrontal cortex, bilateral inferior frontal operculum, left inferior/middle temporal gyrus, and right precentral gyrus (Fig.1A). In contrast, reduced CBF was only detected in the dorsal anterior cingulate cortex (dACC) in ASD versus TD. Clusters showing significant group differences in resting CBF are listed in Tables 2002&^2014^. There were no significant differences in global GM-CBF between ASD (54.48 ± 8.30 mL/100 g/min) and TD (51.35 ± 7.68 mL/100 g/min) children (*t* = 1.22; *P* = n.s.).

**Table 2:** List of cluster showing group differences between autism spectrum disorders (ASD) and typically developing (TD) in cerebral blood flow (CBF) for the whole-brain analysis. Statistical two-sample two-sided *t*-tests were thresholded at a significance level of *t* > 1.69 *P* < 0.05 (type I error corrected at α < 0.05 using cluster size of 251)

	Peak coordinate			#Voxels	Peak T	Anatomical Area	Brodmann area
	*x*	*y*	*z*				
ASD > TD	−6	56	−24	2997	3.50	Medial orbitofrontal cortex	11
	40	10	26	268	3.46	Inferior frontal operculum right	45
	−42	−62	8	523	2.92	Middle temporal gyrus left	21
	−62	0	−10	1085	3.67	Middle temporal gyrus left	21
						Inferior frontal operculum left	45
	−66	−42	−16	496	3.46	Inferior temporal gyrus left	20
	50	−10	48	476	3.11	Precentral gyrus right	4
TD > ASD	6	32	32	392	−2.89	Anterior cingulate cortex	32

**Table 3:** List of cluster showing group differences between autism spectrum disorders (ASD) and typically developing (TD) in functional connectivity (FC) and cerebral blood flow (CBF) within the default mode network (DMN). Statistical two-sample two-sided *t*-tests were thresholded at a significance level of *t* > 1.69 *P* < 0.05 (type I error corrected at α < 0.05 using cluster size of 260 for FC and 251 for CBF)

	FC differences within DMN areas	CBF differences within DMN areas
ASD > TD		
Cluster size (voxels)	574	537
Peak MNI coordinate [*x, y, z*]	6, 40, 24	−6, 56, −24
Peak intensity [*t*-value]	3.80	3.50
Cortical area	Anterior cingulate cortex right	Medial orbitofrontal cortex left
Brodmann area	32/9	11
Cluster size (voxels)		331
Peak MNI coordinate [*x, y, z*]		24, 68, 4
Peak intensity [*t*-value]		3.26
Cortical area		Superior frontal gyrus right
Brodmann area		10
TD > ASD		
Cluster size (voxels)	461	255
Peak MNI coordinate [*x, y, z*]	−10, −48, 54	6, 32, 34
Peak intensity [*t*-value]	−3.77	−2.79
Cortical area	Precuneus/Posterior cingulate cortex left	Anterior cingulate cortex right
Brodmann area	31/7	32

### Perfusion-based FC differences between ASD and TD

ICA decomposed the CBF time series data into 19 group components based on the MDL/BIC model order estimation. We identified two components representing the anterior and posterior part of the DMN (correlation with template RSNs: aDMN = 0.45; pDMN = 0.20, Fig.1B), suggesting that perfusion based FC analysis is able to identify the DMN similar to the networks known from BOLD rs-fMRI. Splitting of the DMN into subnetworks is a common finding (Damoiseaux et al. 2006; Assaf et al. 2010; Washington et al. 2014) and is suggested to represent separate, interacting modules for different cognitive processes (Buckner et al. 2008). Within the DMN, we found increased local FC in the dorsal part of the anterior cingulate cortex (dACC) in children with ASD as compared to the matched TD group. In addition, we found decreased FC between dACC and posterior nodes of the DMN: the precuneus and posterior cingulate cortex (PCC) (Fig.1C, Table 2014). Reduced anterior posterior connectivity was confirmed by seed-based analysis (SBA). Connectivity from these atlas-based ROIs in anterior and posterior areas of the DMN showed significantly reduced values in the ASD group as compared to the TD group: combined posterior ROIs to the dorsal ACC/mPFC (mean FC_ASD_ = 0.36, mean FC_TD_ = 0.56; *t*(df = 37) = −2.4340; *P* < 0.02). The same was true for the FC from each posterior ROI to dorsal ACC/mPFC (*t* = −2.3594 *P* < 0.024/*t* = −2.7418 *P* < 0.01/*t* = −2.1430 *P* < 0.04).

### Region of interest results of CBF and FC

**Figure 2:**
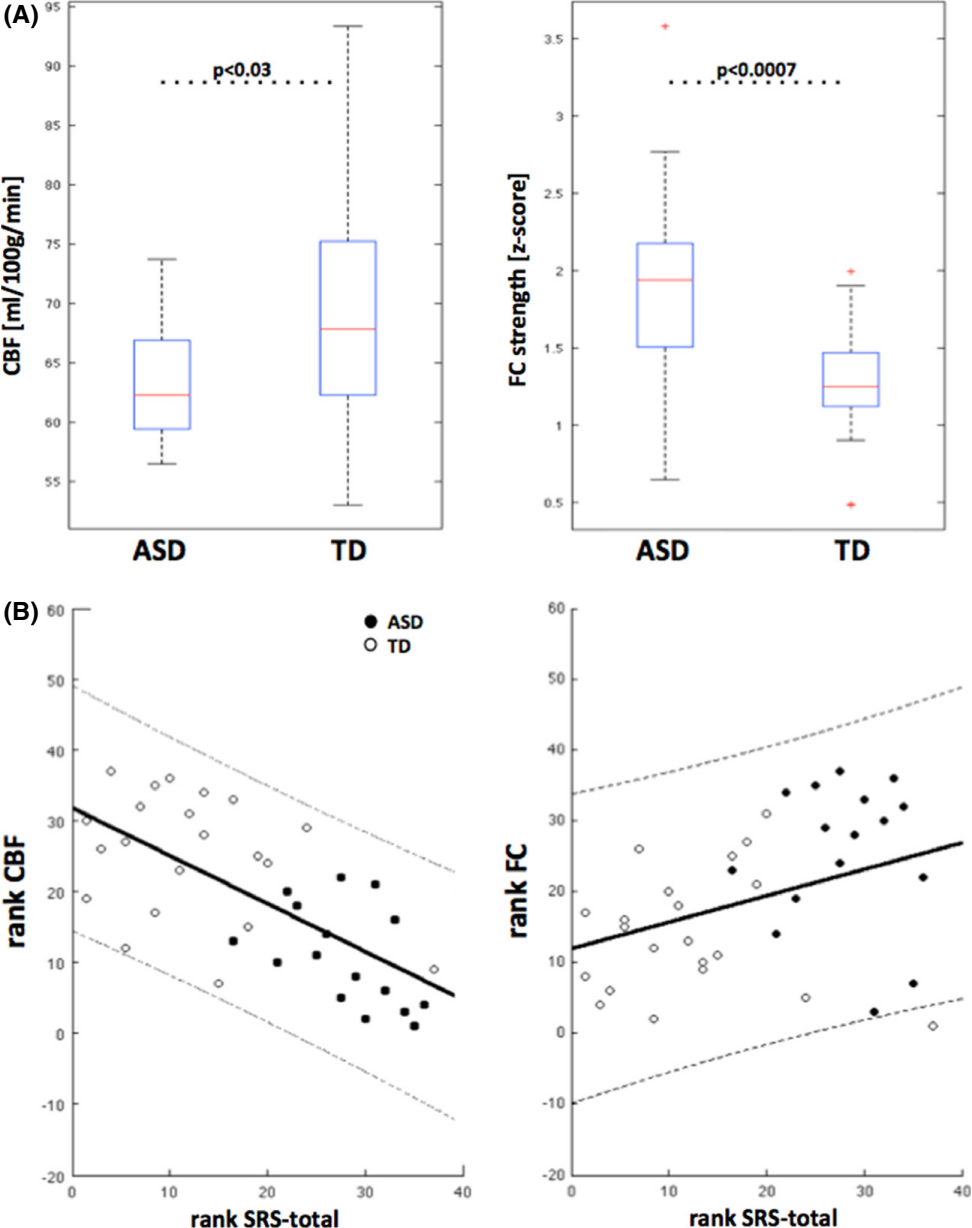
(A) Region of interest (ROI) analysis within the dorsal anterior cingulate cortex (dACC) and group differences between autism spectrum disorders (ASD) and typically developing (TD) displayed as boxplot. ASD exhibits increased functional connectivity (FC) along with reduced cerebral blood flow (CBF) in this specific area. (B) Region of interest (ROI) analysis within the dACC and association to disease severity: Spearman’s rank correlations between Social Responsiveness Scale (SRS)-total scores and functional connectivity (FC) and CBF, respectively. CBF was corrected for global GM-CBF and age (empty circles: TD/filled circles: ASD/line and dashed lines: linear fit and 95% confidence boundaries). There is a significant relation between SRS scores and FC as well as CBF, however, with opposite sign.

Mean CBF and FC values were extracted from ROIs demonstrating significant differences between ASD and TD groups, and were correlated with ASD symptom severity scores. We found significant correlations between FC and CBF with SRS-total T scores in the dACC ROI. Specifically, SRS-total T scores showed a significant positive (*r* = 0.37, *P* < 0.025) correlation with FC, and a negative correlation (*r* = −0.352, *P* < 0.04) with CBF (Fig.2). Accounting for potential structural variations in CBF, by including Jacobians as a covariate, did not alter the negative correlation with SRS-total T scores (*r* = −0.347, *P* < 0.04).

**Figure 3:**
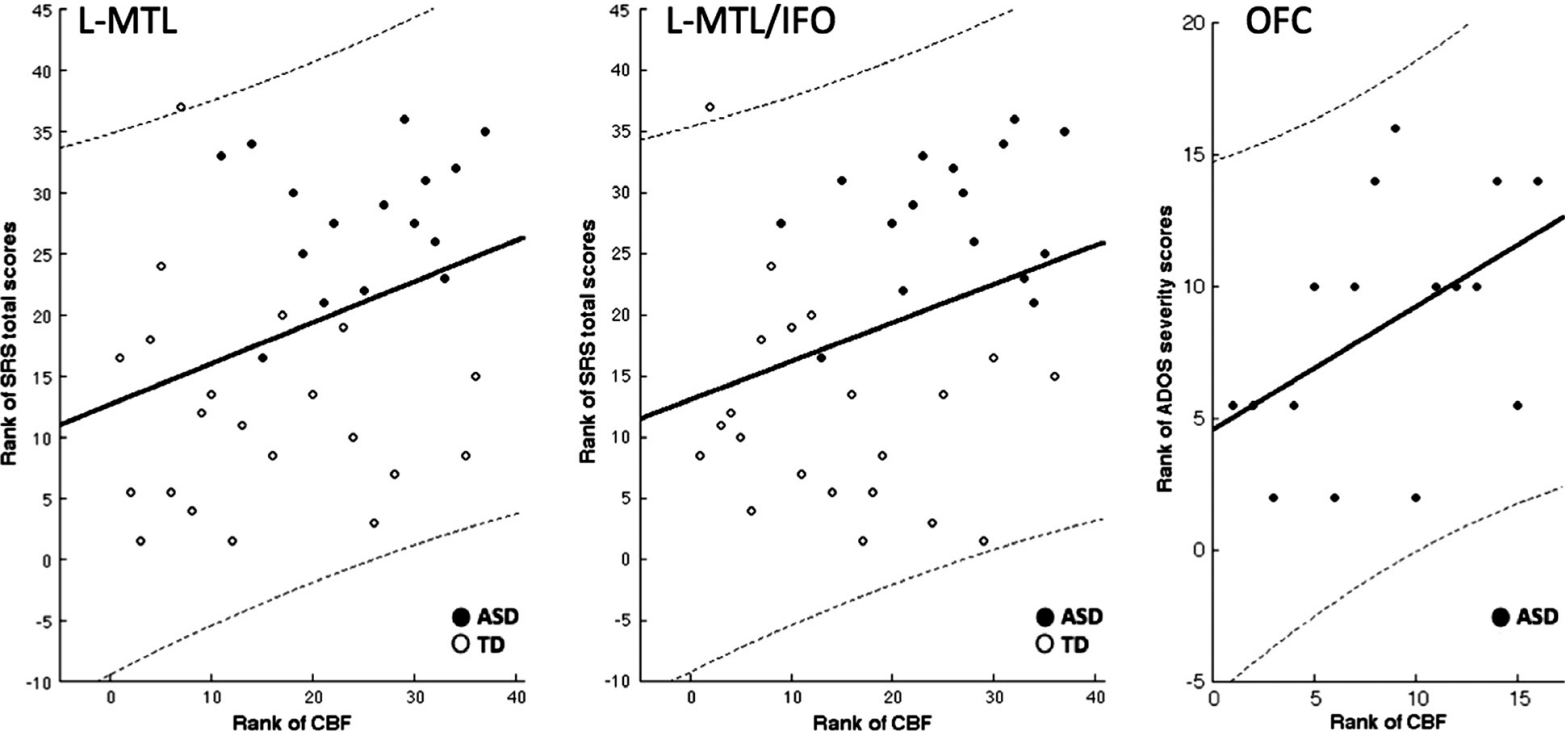
Associations between areas with hyperperfusion in autism spectrum disorders (ASD) and symptom severity scores. Spearman’s rank correlations between Social Responsiveness Scale (SRS)-total scores, respectively, Autism Diagnostic Observation Schedule (ADOS) severity scores to CBF (corrected for global GM-CBF and age). L-MTL: left middle temporal gyrus/L-MTL/IFO: left middle temporal gyrus/inferior frontal operculum/OFC: orbitofrontal cortex.

Within the frontotemporal regions showing hyperperfusion in the ASD group (Fig.3), SRS-total T scores were positively correlated with CBF in the left middle temporal gyrus (*r* = 0.33 *P* < 0.05), inferior temporal gyrus (*r* = 0.34 *P* < 0.04), and inferior frontal operculum (*r* = 0.34 *P* < 0.04) within the whole cohort of ASD and TD children. These correlations with SRS-total T scores, however, did not reach significance when separating the individual ASD or TD groups, suggesting they might have been driven by group differences in social functioning. Finally, CBF in medial orbitofrontal cortex was positively correlated with ADOS severity scores in children with ASD (*r* = 0.49, *P* < 0.05). All above correlations survived correction of structural variations by including Jacobian determinant as a covariate.

### Relationship between FC strength and regional CBF

**Figure 4:**
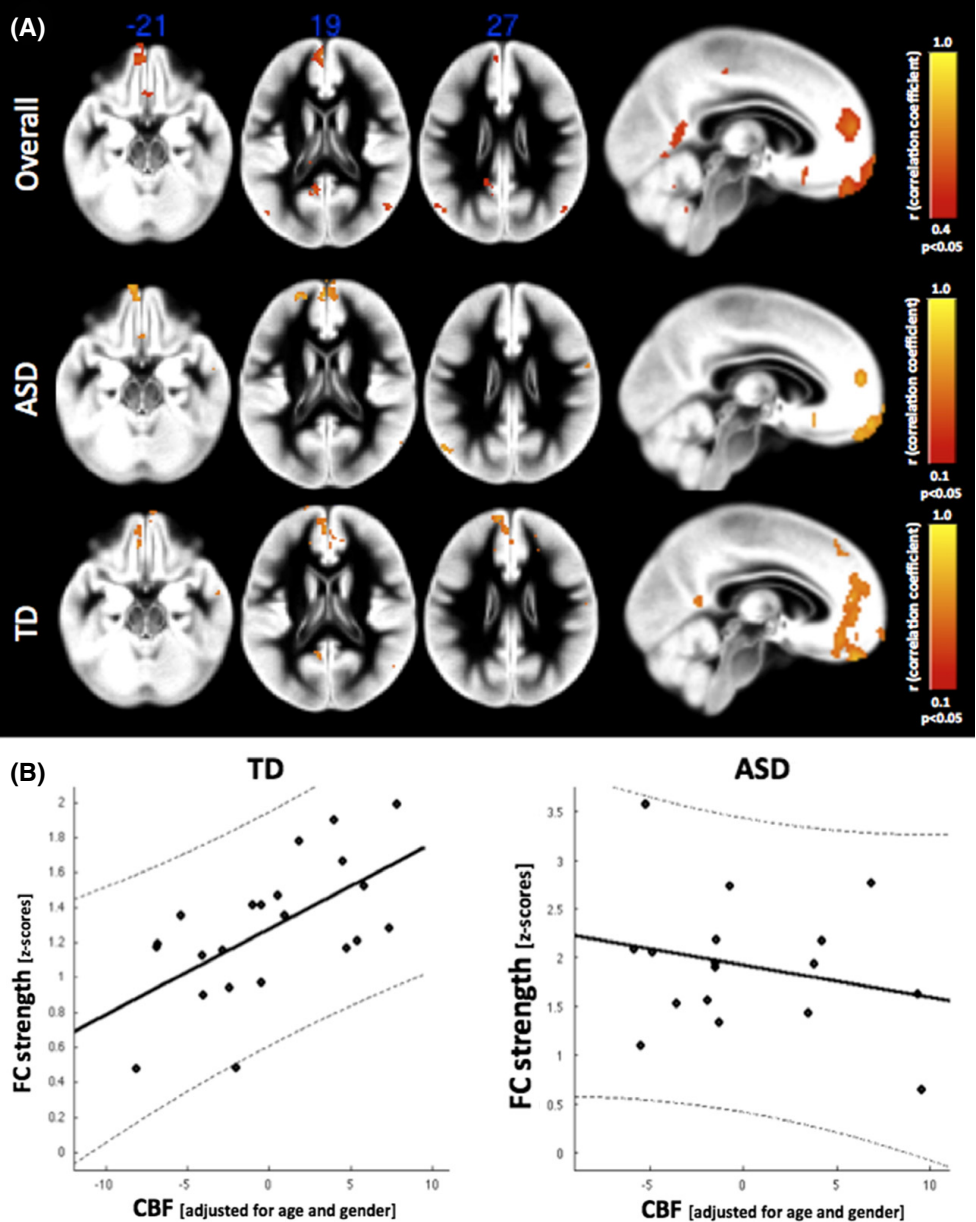
(A) Voxel-wise correlations between functional connectivity (FC) strength (z-scores) and regional cerebral blood flow (CBF) across the combined cohort (autism spectrum disorders (ASD) and typically developing (TD)), as well as in ASD and TD groups, respectively. Correlation patterns revealed a positive association of both physiological characteristics of the brain’s baseline state within the nodes of the default mode network (DMN), however, with a markedly less widespread pattern in the medial frontal cortex of children with ASD. (B) Region of interest (ROI) analysis within the dorsal anterior cingulate cortex (dACC): partial-correlations between functional connectivity (FC) and CBF using globalGM-CBF and age as covariates. TD shows a significant association between CBF and FC strength, whereas this relation is disrupted in ASD.

Using voxel-wise correlation between regional CBF and FC, we found significant correlations (*r* > 0.4, *P* < 0.05, corrected with CST 50) between FC strength (i.e., *z*-scores) and regional CBF in areas of the DMN (Fig.4A top panel), suggesting a positive association between these two physiologic parameters of baseline brain function. Correlation maps for the two groups showed a similar pattern; however, the spatial extent of correlations in the medial frontal cortex in children with ASD was markedly smaller compared to TD children (Fig.4A middle and lower panel; *r* > 0.1, *P* < 0.05).

In particular, the dorsal ACC ROI showed increased local FC and reduced CBF in children with ASD. While voxel-wise analyses across all participants showed a positive association between FC and CBF in this ROI, separate group analyses revealed that this association was present in the TD (*r* = 0.62, *P* < 0.005) but not the ASD group (*r* = −0.08, *P* = n.s.) (Fig.4B). Using Fisher’s *z* transformation to compare the Pearson correlation coefficients between the ASD and TD groups yielded a statistically significant difference (*z* = 2.29; *P* < 0.03). This observation did not change when taking into account possible structural variations: TD (*r* = 0.62, *P* < 0.005); ASD (*r* = −0.21, *P* = n.s.); Fisher’s *z* = 2.63, *P* < 0.005.

## Discussion

In this study, we investigated differences between children with ASD and matched TD children in two aspects of resting brain function: cerebral blood flow (CBF) as a surrogate of basal metabolic activity and functional connectivity (FC) of the Default Mode Network (DMN). While PET/SPECT imaging is typically used to assess CMRglu or CBF and BOLD rs-fMRI to estimate FC, here we capitalized on the ability of the latest pCASL with 3D BS GRASE to provide not only robust CBF measurements but also adequate temporal resolution and SNR for FC analysis (Jann et al. 2015a). To our knowledge, this is the first study to apply ASL to jointly assess CBF and FC in ASD.

### Resting CBF differences between ASD and TD children

The literature on perfusion in ASD is sparse and findings are often discordant, which may be attributed to small sample sizes due to the use of radioactive tracers, the considerable phenotypic heterogeneity seen in individuals with ASD, as well as poorly matched control subjects (Ohnishi et al. 2000). Children undergoing PET/SPECT procedures are commonly sedated, further complicating the interpretation of CBF results. In this study, we employed a state-of-the-art pCASL sequence with single-shot 3D BS GRASE readout to provide robust voxel-wise quantitative CBF values with established accuracy and longitudinal repeatability in the pediatric population (Jain et al. 2012). We also carefully matched the ASD and TD groups in terms of age, gender, and IQ. While we did not find group differences in global mean CBF as has been reported in PET/SPECT (Boddaert and Zilbovicius 2002), we did observe widespread frontotemporal hyperperfusion suggesting hypermetabolism in ASD. Developmental imaging studies in typically developing children using PET and, more recently, ASL have demonstrated an age-related increase in CBF from neonates to toddlers, followed by tapering of CBF from childhood to young adulthood (Chiron et al. 1992; Takahashi et al. 1999; Taki et al. 2011). Notably, there are considerable regional variations of CBF with a posterior to anterior developmental trajectory whereby posterior areas mature earlier than central, temporal, and lastly frontal cortices (Taki et al. 2011; Avants et al. 2015). While there is a general trend of decreasing CBF with age from childhood through adolescence, within the age range of the present cohort (7 to 17 years), aberrant neurodevelopment can manifest as either increased or decreased CBF depending on the developmental trajectory of the particular brain regions of interest. Therefore, the observed widespread frontotemporal hyperperfusion may be interpreted as delayed neurodevelopment in these brain regions in ASD compared to TD (Taki et al. 2011). This observation is consistent with structural MRI findings of enlarged brain size and an overabundance of neurons in the early stages of development, particularly in frontal cortex, of children with ASD (Carper and Courchesne 2005; Courchesne and Pierce 2005a). It has been postulated that the pruning of synapses that normally occurs during later stages of neuronal development is compromised in ASD. Our observation of frontotemporal hyperperfusion is also in accordance with recent MR spectroscopy (MRS) and SPECT findings of reduced GABA concentration and receptor binding in the frontal, temporal (auditory), and motor cortices of children with ASD (Harada et al. 2011; Mori et al. 2012; Gaetz et al. 2014; Rojas et al. 2014). As the primary inhibitory neurotransmitter, GABA concentration has been shown to inversely correlate with CBF by recent studies employing both pCASL and MRS in young healthy volunteers (Donahue et al. 2014; Krause et al. 2014). Our resting perfusion data, in conjunction with structural MRI and MRS findings, suggests a delayed developmental trajectory of lateral and inferior frontal and temporal cortices in children with ASD, potentially characterized by increased regional CBF, compromised synaptic pruning, and reduced GABA concentration.

Notably, perfusion in left temporal and inferior frontal areas, regions commonly associated with language and social function, showed a positive correlation with the level of social impairment across the whole cohort of ASD and TD children. Altered baseline perfusion in these systems could represent a possible mechanism leading to impaired communication and the social deficits observed in ASD. Furthermore, CBF in medial orbitofrontal cortex (OFC) revealed a positive association with ADOS severity in youth with ASD. The OFC has extensive connections to the limbic system and is involved in emotional regulation and decision making. In individuals with ASD, connections between OFC and the limbic system, particularly the amygdale, show developmental alterations, which may be related to impairments in socio-emotional cognition (Bachevalier and Loveland 2006). Taken together, these findings demonstrate that ASL-based perfusion measurements have the potential to elucidate the neurophysiological underpinnings of symptomatology in individuals with ASD.

In addition to frontotemporal hyperperfusion, we found a significant reduction in CBF in the dorsal ACC in children with ASD. One recent SPECT study reported that CBF in the medial prefrontal cortex and ACC is associated with impairments in communication and social interactions (Ohnishi et al. 2000). The exact nature of reduced CBF in dACC in ASD children is presently poorly understood. However, there is evidence that frontal midline areas show a rather constant CBF trajectory from adolescence to adulthood after a peek in childhood (Takahashi et al. 1999; Taki et al. 2011). This is in contrast to lateral and inferior frontal areas where a significant decrease in CBF can be observed during adolescence, as discussed above (Taki et al. 2011). Accordingly, the observed relative hyperperfusion in lateral and inferior frontal areas may be attributed to delayed neurodevelopment in ASD. With regard to the dACC, while there is evidence for gray matter overgrowth in the dACC in autism (Hua et al. 2013), we accounted for potential gray matter differences by including the Jacobian determinant into our CBF analysis. Thus, the mechanisms underlying the observed hypoperfusion in the dACC in ASD remain to be determined. Nevertheless, our ROI analysis showed that lower CBF in the dACC is associated with greater deficits on the Social Responsiveness Scale (SRS; (American Psychiatric Association, 2013)), which is consistent with the SPECT study by Ohnishi et al. (Ohnishi et al. 2000). In addition the dACC is a key node in a network of brain regions thought to underlie self-referential thoughts, social, and emotional processing as well as ToM. Accordingly altered activity of the ACC and impaired communication with other brain areas as found in ASD (Minshew and Keller 2010; Hernandez et al. 2014; Maximo et al. 2014) can lead to impairments in these cognitive processes.

### Functional connectivity in the DMN

Aside from providing quantitative measures of CBF, ASL has recently been demonstrated to reliably identify functionally connected resting brain networks similar to those found in BOLD fcMRI (Jann et al. 2015a). The group ICA approach extracted two components constituting the DMN. It is a well-documented observation that anterior and posterior modules of the DMN show independent behavior and are often split into subnetworks in ICA analyses (Damoiseaux et al. 2006; Esposito et al. 2006; Starck et al. 2013). There are several potential reasons for this: ICA model order influences the decomposition into different network configurations and can result in splitting or merging of network modules (Kiviniemi et al. 2009; Abou-Elseoud et al. 2010; Starck et al. 2013). In particular, splitting of network modules is more frequently observed in cases where connectivity between the modules is reduced, such as in ASD (Assaf et al. 2010; Starck et al. 2013; Washington et al. 2014). There is also ample evidence that the DMN undergoes maturation from childhood to adulthood: while in childhood the DMN is only sparsely connected (i.e., fragmented) it matures and becomes significantly more integrated during adolescence to adulthood (Fair et al. 2007, 2008; Supekar et al. 2010; Uddin et al. 2011; Sherman et al. 2014).

The finding of increased FC in the dACC located within the anterior DMN component indicates a local hyperconnectivity within the frontal lobe in children with ASD, whereas the decreased FC strength with the PCC suggests reduced long-range connectivity between anterior and posterior DMN nodes. This finding of reduced connectivity between anterior and posterior DMN regions was further corroborated by secondary seed based analyses. While reduced internodal long-range FC and segregation of anterior and posterior DMN modules is well in line with findings from BOLD fcMRI (Monk et al. 2009; Assaf et al. 2010; Rudie et al. 2012a; Washington et al. 2014), intranodal short-range hyperconnectivity is somewhat controversial (Rudie and Dapretto 2013; Hernandez et al. 2014). While Assaf et al. (Assaf et al. 2010) found decreased FC in ASD in the ACC within a frontal DMN ICA component, Washington et al. (Washington et al. 2014) reported increased FC of the dACC within the frontal DMN. Furthermore, the latter study presented evidence for a developmental trajectory (Washington et al. 2014) whereby hyperconnectivity is more likely to be found in younger children with ASD than in adolescents or adults with ASD (Uddin et al. 2013b). This developmental model of functional hyper- and hypoconnectivity is likely related to structural alterations at the cellular level during neurodevelopment (i.e., fewer large axons emerging from ACC and connecting to posterior brain areas and excessive number of local connections; (Zikopoulos and Barbas 2010)) and could cause behavioral impairments due to disruption of normal integration of information across brain systems (Courchesne and Pierce 2005b). Accordingly, in individuals with ASD it seems that the frontal cortex may lack feedback from associative brain areas located in posterior brain regions, thus leading to behavioral impairments such as reduced social responsiveness. This notion is corroborated by our finding that increased dACC FC was associated with greater symptom severity as assessed by the SRS. Interestingly, group differences were observed in both resting-state CBF and resting-state FC in the same ROI, dACC. This suggests a possible interaction between these two physiological measures of baseline brain function in the pathophysiology of ASD.

### Relationship between FC and CBF

Comparison of FC to CBF across the combined cohort of ASD and TD children revealed a positive association between these two parameters of resting brain function. Brain organization is determined by trade-offs between the architecture of the cortex, its metabolic operating cost, and the mechanism by which the cortex processes and stores information (Bullmore and Sporns 2012). Computational models suggest that local neuronal activity and their signaling through structural connections give rise to the large-scale functionally connected networks observed in BOLD (Deco et al. 2011, 2013). Neuronal signaling is supported by metabolizing oxygen and glucose, compounds that are delivered by cerebral blood flow. Accordingly, CBF and BOLD signal fluctuations are coupled to neuronal metabolism. Furthermore, in neurodevelopment the architecture of the brain has been found to follow a cost efficient wiring pattern that maximizes functionality with minimal energy consumption (Deco et al. 2011, 2013). Recent reports suggest that the relationship between regional FC and CBF represents the minimum metabolic demand to efficiently process information (Tomasi et al. 2013; Riedl et al. 2014; Passow et al. 2015) and that increased FC requires increased metabolic demand in healthy subjects (Liang et al. 2013; Tomasi et al. 2013).

Our findings suggest that, for the dACC, this relation is intact in healthy children but disrupted in ASD. As discussed above, the dACC has been shown to have altered long- and short-range projections in children diagnosed with ASD. This will be accompanied by a change in metabolic cost as the FC-CBF association has been shown to be stronger for long-range connections (Liang et al. 2013). A neurodevelopmental hypothesis of ASD posits that an alteration in inhibition and excitation in local neuronal clusters in the frontal cortex (Rubenstein and Merzenich 2003; Rojas et al. 2014) leads to cortical malformation (i.e., functionally disorganized and unselective minicolumns (Hussman 2001; Rubenstein and Merzenich 2003)). As inhibition is less energy demanding (Waldvogel et al. 2000), this again suggests that the rules and trade-offs between metabolism and functional connectivity during network development may be altered. Moreover, lack of inhibition can facilitate widespread synchronicity in local connections but impair the establishment of long-range association pathways that are critically dependent on exact timing of neuronal firing patterns during later stages of neurodevelopment (Courchesne and Pierce 2005b; Uhlhaas et al. 2010). Indeed, altered network architectures have been reported in children with ASD, suggesting a loss of integration between distant brain areas. Starck et al. (Starck et al. 2013) showed that in ASD local connectivity in nodes of the DMN seems unaltered, whereas a disruption between anterior and posterior modules has been observed. Supporting evidence for missing or reduced long-range projections is also provided by histological findings in the autistic brain. It was found that in individuals with ASD the number of large axons connecting ACC with posterior brain areas is reduced, whereas excessive local connections through thin axons were present (Zikopoulos and Barbas 2010). Moreover, brain areas with a high degrees of connectivity and long-range projections represent the nodes of connector hubs. As connector hubs, such as the dACC (van den Heuvel and Sporns 2011) are energetically expensive (Karbowski 2007), they are also vulnerable to changes in metabolism and neurovascular coupling. Consequently, they are often affected in many neurological disorders as observed in this study (Ray et al. 2014).

In summary, changes in excitation and inhibition are capable of shifting the balance between factors that define the metabolic cost of brain networks as well as the formation of axonal connections. This can lead to a different pattern of functional connectivity, and consequently regional changes in the normal FC – metabolism coupling, as observed in this study. However, further research is needed to elucidate the mechanism underlying this altered coupling, which might also be present in other developmental or psychiatric disorders. Nevertheless, the use of ASL-based FC and its capability to combine CBF and FC measures provides a valuable tool to investigate the relation between energetic cost and brain network organization.

### Study limitations

The observed pattern of local hyper- and long-range hypoconnectivity is often reported in patient groups as compared to healthy controls. However, it has been found that motion-related artifacts in the BOLD signal may inflate local FC and deflate long-range FC, which may be artificially caused by a greater amount of motion in patient groups (Power et al. 2012). The theory is that spin history is altered due to motion in the magnetic field and the steady-state magnetization is disturbed which leads to changes in image signal intensity (Friston et al. 1996; Muresan et al. 2005). This poses a bias for correlation values between voxels as computed by functional connectivity analyses. Importantly, several studies in children with ASD have demonstrated that these patterns hold after taking into account motion effects (Maximo et al. 2013; Starck et al. 2013; Uddin et al. 2013a). In this study, we addressed this potential confounding factor by comparing motion parameters across groups and also assessing whether there was a relation between the FC estimates and motion parameters (see Supplemental Materials). These analyses showed that our groups were well matched for motion and that there was no evidence of an association between the FC in dACC and the amount of head motion. By employing background suppression (in our sequence to 15% of original strength), the effects of head motion and physiological noise such as cardiac and respiratory pulsation are expected to be reduced as they are proportional or related to raw ASL image intensity (Vidorreta et al. 2013). However, the effects of head motion on perfusion-based FC analysis are not well understood and have just started to be a subject of investigation (Jann et al. 2015b). Another possible source of bias is structural differences in brain size between the ASD and TD groups. We tried to minimize such bias by using an optimized template brain derived from the study cohort. We further used ANTs (Advanced Normalization Tool) to normalize individual subjects to the template brain. Finally, the Jacobian determinants derived from the warp-fields were included as covariates in the ROI analyses to ensure that the presented findings are not due to differences in regional brain volume.

### Summary

This study employed pCASL with 3D BS GRASE to simultaneously investigate aberrant patterns of resting perfusion and FC of the DMN in children with ASD as compared to age, gender and IQ matched TD children. We found widespread hyperperfusion in frontal and temporal cortices as well as hypoperfusion in the dorsal ACC of children with ASD. Changes in connectivity of the DMN were also detected and characterized by locally increased FC within the dorsal ACC and reduced long-range FC between anterior and posterior modules of the DMN. This segregation within DMN modules may cause disturbances in information integration from posterior and anterior cortex and impairments in shifting from internal to external attentional focus. These aberrant patterns of brain activity may help explain impairments in responsiveness and communication, which are linked to functions of the DMN such as ToM and joint attention (Mundy 2003). This was seen in the observed correlations between FC, as well as CBF, and impairments in social responsiveness. As ASL is entirely noninvasive and provides absolute CBF measurement with high test–retest reliability, it may provide an important imaging marker for evaluating treatment effects. ASL may also further inform the pathophysiology as well as the classification of endophenotypes for different subgroups within this complex spectrum of disorders.

## Supporting Information

**Data S1.** Supplemental material providing the A) statistical analyses for motion parameters between ASD and TD groups and B) seed based functional connectivity results between anterior and posterior DMN modules. **Table S1.** ANOVA results for MOTION PARAMETERS between TD and ASD. **Table S2.** Relation between FC in dACC and motion parameters (three translations and three rotations, their first derivatives and the total Framewise Displacement (FD). **Table S3.** ROI MNI coordinates and identification number from atlas (Power et al. 2011). **Figure S1.** Blue spheres indicate location of four ROIs based on Power atlas (4), one in the anterior module, three in the posterior module of the DMN (whereof two also partially overlap with the posterior node identified in the IC component representing the anterior module).
