# Supplementary material for: Altered resting perfusion and functional connectivity of default mode network in youth with autism spectrum disorder
Source: Brain Behav. 2015 Jun 25;5(9):e00358. doi: 10.1002/brb3.358 (PMC4589806; doi:10.1002/brb3.358)
Supplement: Supplementary file 1 [file brb30005-e00358-sd1.docx]

**SUPPLEMENTAL MATERIAL**

We calculated translational and rotational motion for all subjects in control and label images respectively ([1](#_ENREF_1)) and used ANOVA to assess possible differences in motion between the groups that could affect functional connectivity measures. The formula used for the volume-to-volume translational motion is:

 [1]

The rotational motion was calculated using the Euler Angle (EA) and then averaging the absolute value of this angle ([2](#_ENREF_2)) for either label or control images:

 [2]

The total framewise displacement (FD) integrating translational and rotational motion was also computed ([3](#_ENREF_3)). Therefore, rotational motion was recomputed into displacement on the scull surface assuming a radius of 50mm.

a_ij_ = 50 * | radian(α_j_)-radian(α_i_) | [3]

Then FD was defined as:

FD = |x_j_ - x_i_| + |y_j_ - y_i_| + |z_j_ – z_i_| + a_ji_ + b_ji_ +c_ji_ [4]

Where i and j are two consecutive time points, and x, y,z, α, β, and γ are the six parameters of motion (3 translations and 3 rotations) and a, b, c are the surface displacements of α, β, γ.

Then, mean MRD, EA and FD were calculated within each participant to reflect their average amount of volume-to-volume displacement (motion) in translation, rotation or the combined FD during the scan. Finally, these measures were subjected to ANOVAs to test for group differences in motion parameters.

We did not find statistically significant group differences in any motion parameter neither for control nor label images (Table S1).

| **Table S1**  **ANOVA results for MOTION PARAMETERS between TD and ASD** | | | | |
| --- | --- | --- | --- | --- |
|  | **Control images** | | **Label images** | |
|  | **F** | **P** | **F** | **p** |
| **MRD** | 0.16 | 0.69 | 0.1 | 0.75 |
| **EA** | 2.42 | 0.13 | 1.83 | 0.18 |
| **FD** | 0.11 | 0.74 | 0.05 | 0.82 |

In addition, we assessed a possible effect of motion on FC estimates in the dorsal ACC. To this end, we tested if any relation exists between subjects’ FC values (z-scores) and motion parameters (mean translation and rotation as well as their first derivatives, and mean FD) that could indicate such an effect. Our results show that there is no relation between motion parameters and FC strength.

**Table S2**

Relation between FC in dACC and motion parameters (3 translations and 3 rotations, their first derivatives and the total Framewise Displacement (FD). Correlation coefficients (R) and p-values (P) for control (C) and label (L) timeseries.

| **mean** |  |  |  |  |  |  |  |  |  |  |  |  |  |
| --- | --- | --- | --- | --- | --- | --- | --- | --- | --- | --- | --- | --- | --- |
|  | **x** | **y** | **x** | **a** | **b** | **c** | **x'** | **y'** | **x'** | **a'** | **b'** | **c'** | **FD** |
| R (C) | -0.06 | 0.15 | -0.11 | -0.15 | -0.21 | 0.12 | -0.09 | 0.20 | -0.08 | 0.03 | -0.30 | 0.02 | 0.06 |
| P (C) | 0.709 | 0.347 | 0.511 | 0.363 | 0.199 | 0.471 | 0.579 | 0.216 | 0.619 | 0.864 | 0.068 | 0.883 | 0.719 |
| R (L) | 0.00 | 0.09 | -0.09 | -0.14 | -0.18 | 0.16 | -0.08 | 0.16 | -0.09 | 0.03 | -0.24 | 0.01 | 0.06 |
| P (L) | 0.977 | 0.592 | 0.573 | 0.408 | 0.275 | 0.339 | 0.634 | 0.333 | 0.574 | 0.846 | 0.133 | 0.942 | 0.702 |
| **max** |  |  |  |  |  |  |  |  |  |  |  |  |  |
|  | **x** | **y** | **x** | **a** | **b** | **c** | **x'** | **y'** | **x'** | **a'** | **b'** | **c'** | **FD** |
| R (C) | 0.07 | 0.17 | -0.02 | 0.05 | -0.08 | 0.02 | 0.11 | 0.08 | 0.03 | 0.07 | 0.02 | 0.01 | 0.05 |
| P (C) | 0.693 | 0.294 | 0.897 | 0.746 | 0.634 | 0.912 | 0.505 | 0.640 | 0.862 | 0.670 | 0.900 | 0.946 | 0.785 |
| R (L) | 0.08 | 0.15 | 0.05 | 0.03 | -0.04 | 0.06 | 0.12 | 0.07 | 0.19 | 0.08 | 0.17 | 0.00 | 0.14 |
| P (L) | 0.631 | 0.374 | 0.741 | 0.877 | 0.823 | 0.737 | 0.464 | 0.656 | 0.247 | 0.616 | 0.298 | 0.988 | 0.379 |

*
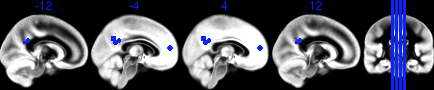
*

*
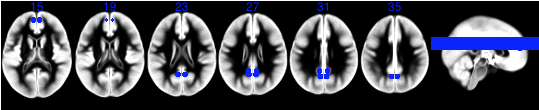
***Supplemental Figure S1:** Blue spheres indicate location of 4 ROIs based on Power atlas ([4](#_ENREF_4)), 1 in the anterior module, 3 in the posterior module of the DMN (whereof two also partially overlap with the posterior node identified in the IC component representing the anterior module).

**Table S3:**

ROI MNI coordinates and identification number from atlas (Power et al Neuron 2011).

| **ROI** | **x** | **y** | **z** |
| --- | --- | --- | --- |
| 88 | -7 | -55 | 27 |
| 89 | 6 | -59 | 35 |
| 92 | 8 | -48 | 31 |
| 105 | 6 | 54 | 16 |

**References**

1. Patriat R*, et al.* (2013) The effect of resting condition on resting-state fMRI reliability and consistency: a comparison between resting with eyes open, closed, and fixated. *NeuroImage* 78:463-473.

2. Van Dijk KR, Sabuncu MR, & Buckner RL (2012) The influence of head motion on intrinsic functional connectivity MRI. *NeuroImage* 59(1):431-438.

3. Power JD, Barnes KA, Snyder AZ, Schlaggar BL, & Petersen SE (2012) Spurious but systematic correlations in functional connectivity MRI networks arise from subject motion. *NeuroImage* 59(3):2142-2154.

4. Power JD*, et al.* (2011) Functional network organization of the human brain. *Neuron* 72(4):665-678.
